# Pathogenic roles of CXCL10 signaling through CXCR3 and TLR4 in macrophages and T cells: relevance for arthritis

**DOI:** 10.1186/s13075-017-1353-6

**Published:** 2017-07-19

**Authors:** Jong-Ho Lee, Bongjun Kim, Won Jong Jin, Hong-Hee Kim, Hyunil Ha, Zang Hee Lee

**Affiliations:** 1 Department of Cell and Developmental Biology, Dental Research Institute, School of Dentistry, Seoul National University, 28 Yeongon-dong, Jongno-gu, Seoul, 110-749 Republic of Korea; 2 Brain Tumor Center and Department of Neuro-Oncology, The University of Texas MD Anderson Cancer Center, Houston, TX 77030 USA; 3 Clinical Research Division, Korean Medicine-Based Herbal Drug Development Group, Korea Institute of Oriental Medicine, 483 Expo-Ro, Yuseong-Gu, Daejeon, 305-811 Republic of Korea

**Keywords:** CXCL10, CXCR3, TLR4, RANKL, Rheumatoid arthritis, Collagen antibody-induced arthritis

## Abstract

**Background:**

Rheumatoid arthritis (RA) is a chronic autoimmune disease characterized by uncontrolled joint inflammation and destruction of bone and cartilage. We previously reported that C-X-C motif chemokine 10 (CXCL10; also called IP-10) has important roles in joint inflammation and bone destruction in arthritis. However, the specific mechanisms by which CXCL10 regulates the recruitment of inflammatory cells and the production of osteoclastogenic cytokines in RA progression are not fully understood.

**Methods:**

Bone marrow-derived macrophages and CD4^+^ T cells were isolated from wild-type (WT), *Cxcl10* ^–/–^, and *Cxcr3* ^–/–^ mice. CXCL10-induced migration was performed using a Boyden chamber, and CXCL10-stimulated production of osteoclastogenic cytokines was measured by quantitative real-time PCR and ELISA. Collagen antibody-induced arthritis (CAIA) was induced by administration of collagen type II antibodies and lipopolysaccharide to the mice. Clinical scores were analyzed and hind paws were collected for high-resolution micro-CT, and histomorphometry. Serum was used to assess bone turnover and levels of osteoclastogenic cytokines.

**Results:**

CXCL10 increased the migration of inflammatory cells through C-X-C chemokine receptor 3 (CXCR3)-mediated, but not toll-like receptor 4 (TLR4)-mediated, ERK activation. Interestingly, both receptors CXCR3 and TLR4 were simultaneously required for CXCL10-stimulated production of osteoclastogenic cytokines in CD4^+^ T cells. Furthermore, calcineurin-dependent NFATc1 activation was essential for CXCL10-induced RANKL expression. In vivo, F4/80^+^ macrophages and CD4^+^ T cells robustly infiltrated into synovium of WT mice with CAIA but were significantly reduced in both *Cxcl10* ^*–/–*^ and *Cxcr3* ^*–/–*^ mice. Serum concentrations of osteoclastogenic cytokines and bone destruction were also reduced in the knockout mice, leading to attenuated progression of arthritis.

**Conclusion:**

These findings highlight the importance of CXCL10 signaling in the pathogenesis of RA and provide previously unidentified details of the mechanisms by which CXCL10 promotes the development of arthritis.

**Electronic supplementary material:**

The online version of this article (doi:10.1186/s13075-017-1353-6) contains supplementary material, which is available to authorized users.

## Background

Osteoclasts, multinucleated bone-resorbing cells, are essential mediators of inflammatory bone erosion and are derived from monocyte/macrophage lineage precursor cells through the action of RANKL, a key factor for osteoclastogenesis [1]. Increased bone resorption by activated osteoclasts can be caused by systemic alterations such as the upregulation of osteotropic or osteoclastogenic cytokines, including RANKL, TNFα, and IL-6, which in turn cause bone-destructive diseases, including rheumatoid arthritis (RA) [2].

RA is a common, chronic inflammatory and systemic autoimmune disease characterized by progressive infiltration of lymphocytes and macrophages into synovium, leading to persistent synovial inflammation and destruction of bone and cartilage [3]. Abnormal activation of T cells, one of the most abundant cell populations in the RA synovium, drives chronic inflammation and joint destruction [4]. The T cells interact with other immune and resident cells including macrophages by secreting cytokines and chemokines or by direct cell-to-cell contact, leading to a boost in production of proinflammatory cytokines and chemokines [5, 6]. The increased cytokines and chemokines in turn alter the activation or differentiation state of one or both of the cell types [4]. In particular, elevated proinflammatory cytokines and chemokines increase the expression of osteoclastogenic cytokines including RANKL, TNFα, and IL-6 from CD4^+^ T cells of RA patients [7–9].

Synovitis, inflammation of a synovial membrane, is initiated by infiltration of inflammatory cells into the synovial compartment, followed by synovial hyperplasia, also called pannus, in RA synovial tissue [3, 10]. The hyperplastic synovial pannus in RA produces proinflammatory cytokines, chemokines, and proteases and subsequently invades and destroys bone and cartilage [10, 11]. Particularly, macrophages and fibroblast-like synoviocytes are central effectors of synovitis and synovial pannus formation, respectively [3, 10]. Thus, infiltration of inflammatory cells into the synovium is an essential step for the progression of RA.

C-X-C motif chemokine 10 (CXCL10), also known as interferon-gamma-inducible protein 10 (IP-10), was initially identified as a chemokine secreted by several cell types including macrophages, endothelial cells, and fibroblasts in response to interferon gamma (IFN-γ) [12]. CXCL10 binds to its receptor CXCR3 and regulates immune responses through recruitment of leukocytes, including T cells and monocytes/macrophages [12]. In addition to its binding to CXCR3, CXCL10 has been shown to bind with and activate toll-like receptor 4 (TLR4) [13]. The crucial role of CXCL10 has been well characterized in chronic Th1 inflammatory diseases. In inflamed tissues, CXCL10 recruits Th1 cells via CXCR3 where IFN-γ is secreted, which in turn stimulates CXCL10 expression in various cell types, resulting in positive feedback to amplify CXCL10 and Th1 responses [14]. It has been shown that CXCL10 levels in synovial fluid and serum are elevated in human RA patients and in mice with collagen-induced arthritis (CIA) [6, 15, 16]. Blockade of the CXCL10–CXCR3 axis has been shown to inhibit the infiltration of inflammatory cells, including T cells and macrophages, into inflamed joints and to decrease the severity of arthritis and bone and cartilage destruction in animal models of RA [6, 17]. In addition to its chemotactic effect, CXCL10 has been shown to increase RANKL expression in CD4^+^ T cells [6, 18]. However, the exact mechanisms by which CXCL10 regulates inflammatory cell infiltration and osteoclastogenic cytokine production in RA are not fully understood.

In general, the CIA model is widely used in animal studies and shares many pathological and histological similarities with RA, which is known to be both T-cell and B-cell dependent. The model is commonly used to evaluate both the antigen-recognition phase and the inflammatory phase of arthritis [19]. However, the susceptibility for CIA is poor in mice of the C57BL/6 background, the strain widely used for knockout models. By contrast, collagen antibody-induced arthritis (CAIA) is induced by infiltration of neutrophils and macrophages into the inflamed joints [20, 21]. Many of the pathological symptoms that are typical of RA can be seen in the joints of animals with CAIA, such as synovitis, pannus formation, and destruction of cartilage and bone [22, 23]. In addition, the CAIA model not only is a suitable model for studies of arthritis-induced osteoporosis [24] but also can be used in genetically modified C57BL/6 mice, such as transgenics and knockouts [22].

In this study, we investigated the functional roles and specific mechanisms of CXCL10 signaling in RA progression using *Cxcl10* ^*–/–*^, *Cxcr3* ^*–/–*^, and *Tlr4* ^*–/–*^ knockout mice.

## Methods

### Animals

Wild-type (WT), *Cxcl10* ^–/–^, and *Cxcr3* ^–/–^ mice (C57BL/6 background) were obtained from The Jackson Laboratory (Bar Harbor, ME, USA). *Tlr4* ^*–/–*^ mice were kindly provided by Dr Sung Joong Lee (Seoul National University, Seoul, Korea). All animal procedures were reviewed and approved by the animal care committee of the Institute of Laboratory Animal Resources of Seoul National University.

### Induction and evaluation of CAIA

Male 8-week-old mice were injected intravenously with a five-clone cocktail of collagen type II antibodies (5 mg/mouse; Chondrex, Redmond, WA, USA) to induce arthritis (CAIA group). Nonarthritic control mice received phosphate-buffered saline (control group). Three days after antibody administration, 100 μg lipopolysaccharide (LPS) was injected intraperitoneally in both CAIA and control mice.

The severity of arthritis was assessed according to paw swelling and was scored on a scale of 0–3 (where 0 = normal, 1 = swelling of the toes, 2 = swelling of the sole of the foot or increased swelling, and 3 = severe swelling or swelling of the entire paw). The arthritic score for each mouse was expressed as the sum of the scores of the four limbs. Mice were sacrificed on day 12, and serum and paws were collected. Paws were fixed in 4% paraformaldehyde overnight and were then washed with PBS. Three-dimensional images of posterior paws were obtained by microfocal computed tomography (micro-CT) scanning (SMX-90CT; Shimadzu, Japan). An eroded bone surface per total bone surface was determined using TRI 3D-BON software (RATOC System Engineering Co., Kyoto, Japan). For histological analysis, posterior paws were decalcified in 12% EDTA and then embedded in paraffin.

### Histological evaluation

Paraffin-embedded sections of decalcified posterior paws were prepared as described previously [25]. The sections were stained with Harris hematoxylin and eosin (H&E), Safranin O and methyl green staining, and tartrate-resistant acid phosphatase (TRAP; Sigma-Aldrich, St Louis, MO, USA) and then scored as described previously [26, 27].

The H&E-stained sections were scored for inflammation and pannus formation. Inflammation was scored according to the following criteria: 0 = normal, 1 = minimal infiltration, 2 = mild infiltration, 3 = moderate infiltration, 4 = marked infiltration, and 5 = severe infiltration. Pannus formation was scored according to the following criteria: 0 = no pannus formation, 1 = minimal pannus formation, 2 = mild pannus formation, 3 = moderate pannus formation, 4 = marked pannus formation, and 5 = severe pannus formation.

The Safranin O-stained sections were scored for cartilage damage. Cartilage damage was scored according to the following criteria: 0 = no damage, 1 = minimal loss of cartilage, 2 = mild loss of cartilage, 3 = moderate loss of cartilage, 4 = marked loss of cartilage, and 5 = severe diffuse loss of cartilage.

The TRAP-stained sections were scored for osteoclast activity. Osteoclast activity was scored according to the following criteria: 0 = no staining, 1 = rare positive cells, 2 = scattered staining, 3 = multiple foci of positive cells, 4 = clusters of positive cells, and 5 = diffuse staining.

### Immunohistochemistry

Immunohistochemical analysis was performed on decalcified paraffin-embedded tissue sections as described previously [28]. The anti-F4/80 antibody clone CI:A3-1 (Abcam, Cambridge, MA, USA) that recognizes the mouse F4/80 antigen, a cell surface glycoprotein expressed at high levels on various murine macrophages, was used to detect macrophages in arthritic joints. Detection of the primary antibody was performed using the VECTASTAIN® Elite ABC kit (Vector Laboratories, Burlingame, CA, USA), followed by 3,3-diaminobenzidine (Vector Laboratories) incubation and nuclear staining with hematoxylin. Six randomly chosen fields per slide were analyzed and averaged.

### Immunohistofluorescence

The deparaffinized sections were treated with a citrate buffer (pH 6.0) for antigen retrieval in a microwave oven for 30 min. After washing, tissue sections were blocked for nonspecific binding with 5% horse serum/0.3% Tween-20/TBS and incubated with FITC-labeled rat anti-mouse CD4 (clone GK1.5, 1:100; eBioscience, San Diego, CA, USA) antibody at 4 °C overnight. The FITC-labeled rat igG2b K isotype control (clone eB149/10H5, 1:100; eBioscience) was used to control staining specificity. After a final washing, cover slips were mounted onto slides with fluoroshield mounting medium with DAPI (four slips were mounted on indole; Abcam). These sections were examined using an LSM 700 confocal laser-scanning microscope (Zeiss, Oberkochen, Germany).

### Isolation of bone marrow-derived macrophages and activated CD4^+^ T cells and gene transduction

Bone marrow-derived macrophages (BMMs) were prepared as described previously [25]. In brief, mouse bone marrow cells were obtained from femurs and tibias and incubated in α-MEM complete media containing 10% fetal bovine serum, 100 units/ml penicillin, and 100 μg/ml streptomycin on 10-cm culture dishes in the presence of M-CSF (10 ng/ml) overnight. Nonadherent cells were transferred to 10-cm bacterial culture dishes and were further cultured in the presence of M-CSF (30 ng/ml) for 3 days. Adherent cells were used as BMMs after the nonadherent cells were washed out.

CD4^+^ T cells were isolated from mouse spleens as described previously [6]. Briefly, spleens were mashed in Hanks’ balanced salt solution containing 3× antibiotics. Cells were harvested, and red blood cells were removed by ammonium–chloride–potassium (ACK) lysing buffer. The remaining cells were collected and separated with a Ficoll-Histopaque (Sigma-Aldrich) discontinuous gradient. The interface containing the cells was washed with PBS, resuspended in RPMI 1640 (Welgene, Daegu, Korea) containing 10% FBS, and then cultured for 1 day. Nonadherent cells were then cultured for 3 days in the presence of 50 ng/ml IL-2 (PeproTech, London, UK). Activated CD4^+^ T cells were purified by negative selection using CD4^+^ T-cell isolation kits (Miltenyi Biotec, Bergisch Gladbach, Germany) according to the manufacturer’s instructions.

CD4^+^ T cells were transduced with the retroviral vectors pMX-IRES-EGFP (control) or pMX-CA-NFATc1-IRES-EGFP encoding a constitutively active (CA) form of NFATc1 as described previously [25]. BMMs were transduced with the adenoviral vectors Ax1w1 (control) or AxMEK^CA^ carrying CA-MEK1 (Ser 218 and Ser222 to Glu) as described previously [29].

### Migration assay

Amounts of 1 × 10^5^ BMMs in 200 μl of serum-free α-MEM were seeded in the upper well of a Boyden chamber with polycarbonate filters containing 8-μm pore membranes (Corning Costar, Cambridge, MA, USA). The lower well was loaded with 600 μl of serum-free α-MEM with or without recombinant mouse CXCL10 (PeproTech). After 12 hours of incubation, cells attached on the lower surface of the membrane were fixed with 100% methanol for 10 min and then stained with Harris H&E. Migrated cells were quantified using an image analysis system (Image J; National Institutes of Health, Bethesda, MD, USA).

Amounts of 5 × 10^5^ CD4^+^ T cells in 200 μl serum-free RPMI 1640 medium were seeded in the upper well of the Boyden chamber with polycarbonate filters containing 5-μm pore membranes. The lower well was loaded with 600 μl serum-free RPMI 1640 medium with or without recombinant mouse CXCL10 and the cells were allowed to migrate for 12 hours. The migrated cells recovered from each bottom well were counted as follows: 100 μl of culture media containing migrated cells was collected from each bottom chamber and counted as migrated cells.

### ELISA

The amount of CXCL10, RANKL, TNFα, IL-6, and C-terminal telopeptides of type I collagen (CTX) in serum was determined using the corresponding ELISA kits (R&D Systems, Minneapolis, MN, USA) according to the manufacturer’s instructions. In some experiments, protein amounts of RANKL and TNFα in CD4^+^ T-cell culture supernatants were determined using the same ELISA kits mentioned earlier.

### Immunoblotting

Immunoblot analysis was performed as described previously [25]. Specific antibodies against NFATc1 (Santa Cruz Biotechnology, Santa Cruz, CA, USA), phospho-ERK1/2 (Thr202/Tyr204) and ERK (Cell Signaling Technology, Danvers, MA, USA), MEK1 (Millipore, Boston, MA, USA), and β-actin (Sigma-Aldrich) were used.

### Reverse transcription and real-time PCR analysis

Total RNA was prepared from cells or spleens using an RNeasy Mini kit (Qiagen, Valencia, CA, USA) according to the manufacturer’s instructions, and cDNA was synthesized from 2 μg of total RNA by reverse transcriptase (Superscript II Preamplification System; Gibco**-**BRL, Gaithersburg, MD, USA). Real-time PCR was performed on an ABI Prism 7500 sequence detection system using a SYBR® Green PCR Master Mix (Applied Biosystems, Foster City, CA, USA) and following the manufacturer’s protocols. The ABI 7500 sequence detector was programmed with the following PCR conditions: 40 cycles of 15-s denaturation at 95 °C and 1-minute amplification at 60 °C. All reactions were run in triplicate and normalized to the housekeeping gene β-actin. The evaluation of relative differences of PCR results was calculated using the comparative cycle threshold (C_T_) method. The following primer sets were used: mouse RANKL forward, 5′-TGGAAGGCTCATGGTTGGAT-3′ and reverse, 5′-CATTGATGGTGAGGTGTGCA-3′; mouse TNFα forward, 5′-GACCCTCACACTCAGATCATCTTCT-3′ and reverse, 5′-CCTCCACTTGGTGGTTTGCT-3′; mouse IL-6 forward, 5′-GTCCTTCCTACCCCAATTTCC A-3′ and reverse, 5′-GGATGGTCTTGGTCCTTAGCC-3′; mouse CXCR3 forward, 5′-CAGCCTGAACTTTGACAGAACCT-3′ and reverse, 5′-GCAGCCCCAGCAAGAAGA-3′; mouse TLR4 forward, 5′-AATCCTCTGGGGAGGCACAT-3′ and reverse, 5′-CAGGTCCAAGTTGCCGTTTC-3′; and mouse β-actin forward, 5′-ATGTGGATCAGCAAGCAGGA-3′ and reverse, 5′-AAGGGTGTAAAACGCAGCTC-3′.

### Statistical analysis

Data are presented as the mean ± SD (in-vitro data) or the mean ± SEM (in-vivo data). Statistical analysis was performed by either unpaired, two-tailed Student’s *t* test, one-way ANOVA followed by Dunnett’s test, or two-way repeated-measures ANOVA followed by Bonferroni’s test using GraphPad Prism 5.0 (GraphPad Software, San Diego, CA, USA). Values of *P* < 0.05 were considered significant.

## Results

### CXCL10 induces migration of inflammatory cells through CXCR3-mediated ERK activation but not TLR4

**Fig. 1:**
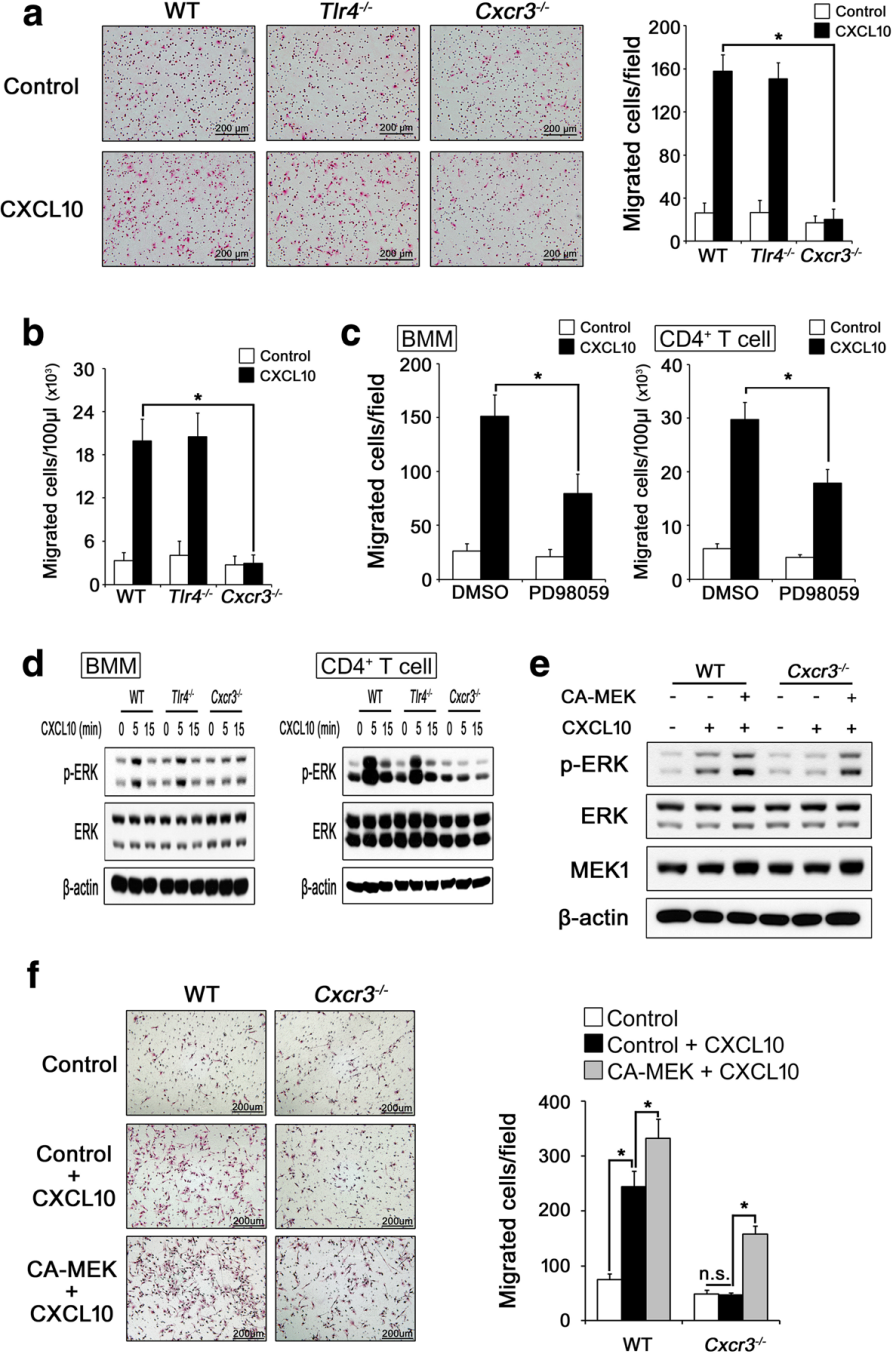
CXCL10 induces migration of BMMs and CD4^+^ T cells through CXCR3 but not TLR4. **a**, **b** BMMs and CD4^+^ T cells were isolated from wild-type (*WT*), *Tlr4* ^*–/–*^, and *Cxcr3* ^*–/–*^ mice and then the cells were serum starved. Cell migration in response to CXCL10 (100 ng/ml) was assessed in transwell chambers for 12 hours. Representative images of migrated BMMs. (**a**, *left panel*; *scale bar*, 200 μm). Number of migrated BMMs (**a**, *right panel*) and CD4^+^ T cells (**b**). **c** Serum-starved BMMs (*left panel*) and CD4^+^ T cells (*right panel*) were preincubated with DMSO or 10 μM PD98059 for 1 hour, and migration was measured in the absence or presence of CXCL10 (100 ng/ml) after 12 hours. **d** Serum-starved BMMs (*left panel*) and CD4^+^ T cells (*right panel*) were stimulated with CXCL10 (100 ng/ml) for the indicated times. Total cell lysates were immunoblotted with the indicated antibodies. **e**, **f** WT or *Cxcr3* ^*–/–*^ BMMs were infected with adenovirus carrying empty vector (Ax1w1) or CA-MEK1 (AxMEK^CA^). After 24 hours, the infected cells were serum starved and stimulated with PBS (vehicle) or CXCL10 (100 ng/ml) for 5 min. Total cell lysates were immunoblotted with the indicated antibodies (**e**). Migration of the infected cells in response to CXCL10 (100 ng/ml) was assessed in transwell chambers for 12 hours. Representative images of migrated BMMs (**f**, *left panel*; *scale bar*, 200 μm) and the number of migrated BMMs (**f**, *right panel*). Results shown are representative of three independent experiments (*n* = 3), and values are expressed as mean ± SD. *P < 0.001 by one-way ANOVA followed by Dunnett’s test. *CXCL10* C-X-C motif chemokine 10, *Tlr4* toll-like receptor 4, *Cxcr3* CXC chemokine receptor 3, *n.s.* nonsignificant, *BMM* bone marrow-derived macrophage

Because CXCL10 has been reported to bind to TLR4 as well as to CXCR3 [13], we first investigated whether TLR4 is involved in CXCL10-induced cell migration. We isolated BMMs and CD4^+^ T cells from WT, *Tlr4* ^*–/–*^, and *Cxcr3* ^*–/–*^ mice. In our experimental condition, CD4^+^ T cells from the WT, *Tlr4* ^*–/–*^, and *Cxcr3* ^*–/–*^ spleens were enriched to about 94% purity by magnetic bead separation (Additional file 1: Figure S1a), and IL-2 treatment successfully increased CXCR3 and IL-2 mRNA expression with similar maintained viability of all CD4^+^ T cells from WT, *Tlr4* ^*–/–*^, and *Cxcr3* ^*–/–*^ mice (Additional file 1: Figure S1b, c). We next carried out chemotactic assay experiments. As expected, the CXCL10-induced migration of BMMs (Fig. 1a and Additional file 1: Figure S2) and CD4^+^ T cells (Fig. 1b and Additional file 1: Figure S3) was blocked in *Cxcr3* ^*–/–*^ or si-CXCR3 cells compared with WT or si-Control cells. TLR4 knockout, however, did not affect CXCL10-induced migration (Fig. 1a, b).

CXCL10 stimulates ERK [6], which positively regulates cell migration [30]. Therefore, we checked whether CXCL10 induces cell migration through the CXCR3–ERK axis. ERK inhibition by PD98059, a specific MEK inhibitor, greatly reduced CXCL10-induced migration of BMMs and CD4^+^ T cells (Fig. 1c and Additional file 1: Figure S4). In addition, CXCR3 deficiency resulted in failed CXCL10-induced ERK phosphorylation in both BMMs and CD4^+^ T cells, whereas TLR4 deficiency did not (Fig. 1d). The inhibition of CXCL10-induced ERK phosphorylation and migration was largely rescued by overexpression of a constitutively active MEK1 in *Cxcr3* ^*–/–*^ cells (Fig. 1e, f). These results suggest that CXCR3-mediated ERK activation is crucial for the CXCL10-induced cell migration.

### CXCL10 induces the production of osteoclastogenic cytokines in CD4^+^ T cells through both TLR4 and CXCR3

**Fig. 2:**
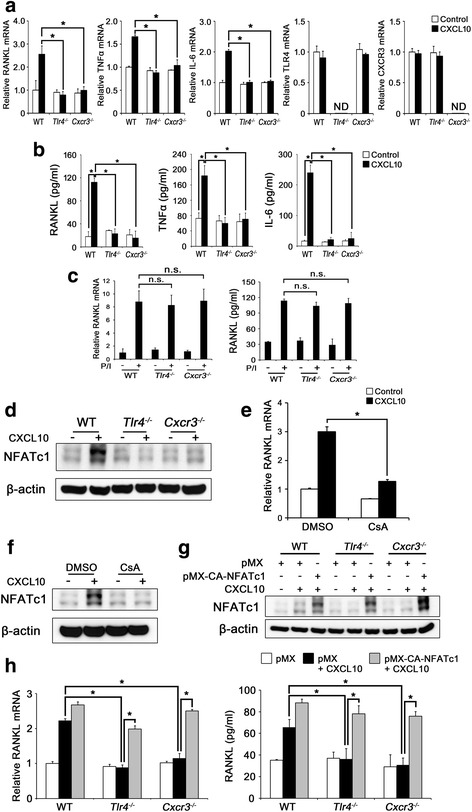
CXCL10 stimulates production of osteoclastogenic cytokines in CD4^+^ T cells through TLR4 and CXCR3. **a**, **b** CD4^+^ T cells were isolated from WT, *Tlr4* ^*–/–*^, and *Cxcr3* ^*–/–*^ mice, and serum-starved CD4^+^ T cells were cultured with or without CXCL10 (100 ng/ml) for 24 hours. RANKL, TNFα, IL-6, CXCR3, and TLR4 mRNA levels were analyzed by real-time PCR (**a**). RANKL, TNFα, and IL-6 protein levels in culture media were quantified by ELISA (**b**). **c** CD4^+^ T cells from WT, *Tlr4* ^*–/–*^, and *Cxcr3* ^*–/–*^ mice were serum starved and treated with or without 500 ng/ml ionomycin and 20 ng/ml PMA (P/I) for 6 hours. RANKL mRNA levels and RANKL protein levels in culture media were measured by real-time PCR (*left panel*) and ELISA (*right panel*), respectively. **d** CD4^+^ T cells were serum starved and treated with or without CXCL10 (100 ng/ml) for 24 hours. Total cell lysates were immunoblotted with the indicated antibodies. **e**, **f** Serum-starved WT CD4^+^ T cells were preincubated with DMSO or 10 nM CsA and then stimulated with or without CXCL10 (100 ng/ml) for 24 hours. RANKL mRNA levels were analyzed by real-time PCR (**e**). NFATc1 protein levels were measured by western blotting (**f**). **g**, **h** CD4^+^ T cells from the indicated mice were transduced with the retroviral vectors pMX-IRES-EGFP (*pMX*) or pMX-CA-NFATc1-IRES-EGFP (*pMX-CA-NFATc1*). The cells were serum starved and cultured with or without CXCL10 (100 ng/ml) for 24 hours. Total cell lysates were immunoblotted with the indicated antibodies (**g**). RANKL mRNA levels (**h**, *left panel*) and RANKL protein levels in culture media (**h**, *right panel*) were measured. Results shown are representative of three independent experiments (*n* = 3), and values are expressed as mean ± SD. *P < 0.001 by one-way ANOVA followed by Dunnett’s test. *CXCL10* C-X-C motif chemokine 10, *RANKL* receptor activator of nuclear factor kappa-B ligand, *TNFα* tumor necrosis factor alpha, *IL-6* interleukin 6, *Tlr4* toll-like receptor 4, *Cxcr3* CXC chemokine receptor 3, *WT* wild-type, *NFATc1* nuclear factor of activated T cells, cytoplasmic 1, *DMSO* dimethyl sulfoxide*, CsA* cyclosporin A, *ND* not detected, *n.s.* nonsignificant

Beyond its chemotactic effect, CXCL10 has been shown to induce osteoclastogenic cytokines [6, 18]. Therefore, we next investigated the functional role of CXCR3 and TLR4 in response to CXCL10 in the expression of osteoclastogenic cytokines. Consistent with our previous report [6], CXCL10 significantly induced RANKL, TNFα, and IL-6 mRNA in CD4^+^ T cells isolated from WT mice, but not in *Cxcr3* ^*–/–*^ CD4^+^ T cells (Fig. 2a). In line with these results, the CXCL10-induced upregulation of RANKL, TNFα, and IL-6 mRNA was greatly inhibited in CXCR3-depleted CD4^+^ T cells (Additional file 1: Figure S3). Interestingly, the inhibition was also found in CD4^+^ T cells isolated from *Tlr4* ^*–/–*^ mice (Fig. 2a). To confirm this result, we measured the amount of RANKL, TNFα, and IL-6 secreted into the culture medium by ELISA, and the results were consistent with the mRNA expression (Fig. 2b). To exclude the possibility that the inhibitory effect was a side effect of a genetic disorder, we treated the CD4^+^ T cells with PMA/ionomycin to induce RANKL expression [31]. As shown in Fig. 2c, PMA/ionomycin-induced expression of both mRNA and protein for RANKL was not significantly different between the cells. Altogether, these results suggest that both CXCR3 and TLR4 are specifically required for CXCL10-stimulated production of osteoclastogenic cytokines.

NFAT-binding sites exist in the RANKL promoter to induce RANKL expression in CD4^+^ T cells, which is regulated by the calcineurin signaling pathway [31, 32]. Moreover, autoamplification of NFATc1 expression is mediated by activated calcineurin-dependent NFATc1 activation [33, 34]. We reported previously that CXCL10 induces NFATc1 expression in CD4^+^ T cells [6], which was abolished in both *Cxcr3* ^*–/–*^ and *Tlr4* ^*–/–*^ CD4^+^ T cells (Fig. 2d). To investigate whether calcineurin signaling plays a critical role in CXCL10-induced RANKL and NFATc1 expression, we performed real-time PCR and western blot analysis on samples prepared in the absence or presence of cyclosporin A (CsA, an inhibitor of calcineurin activity). Compared with the control, CsA pretreatment greatly inhibited CXCL10-induced RANKL (Fig. 2e) and NFATc1 expression (Fig. 2f). Furthermore, overexpression of CA-NFATc1 fully rescued the failed RANKL expression in both *Cxcr3* ^*–/–*^ and *Tlr4* ^*–/–*^ CD4^+^ T cells (Fig. 2g, h). These data indicate that the calcineurin/NFATc1 pathway is essential for CXCL10-induced RANKL expression as well as NFATc1 expression in T cells.

### CAIA progression is ameliorated in *Cxcl10*^*–/–*^ and *Cxcr3*^*–/–*^ mice

**Fig. 3:**
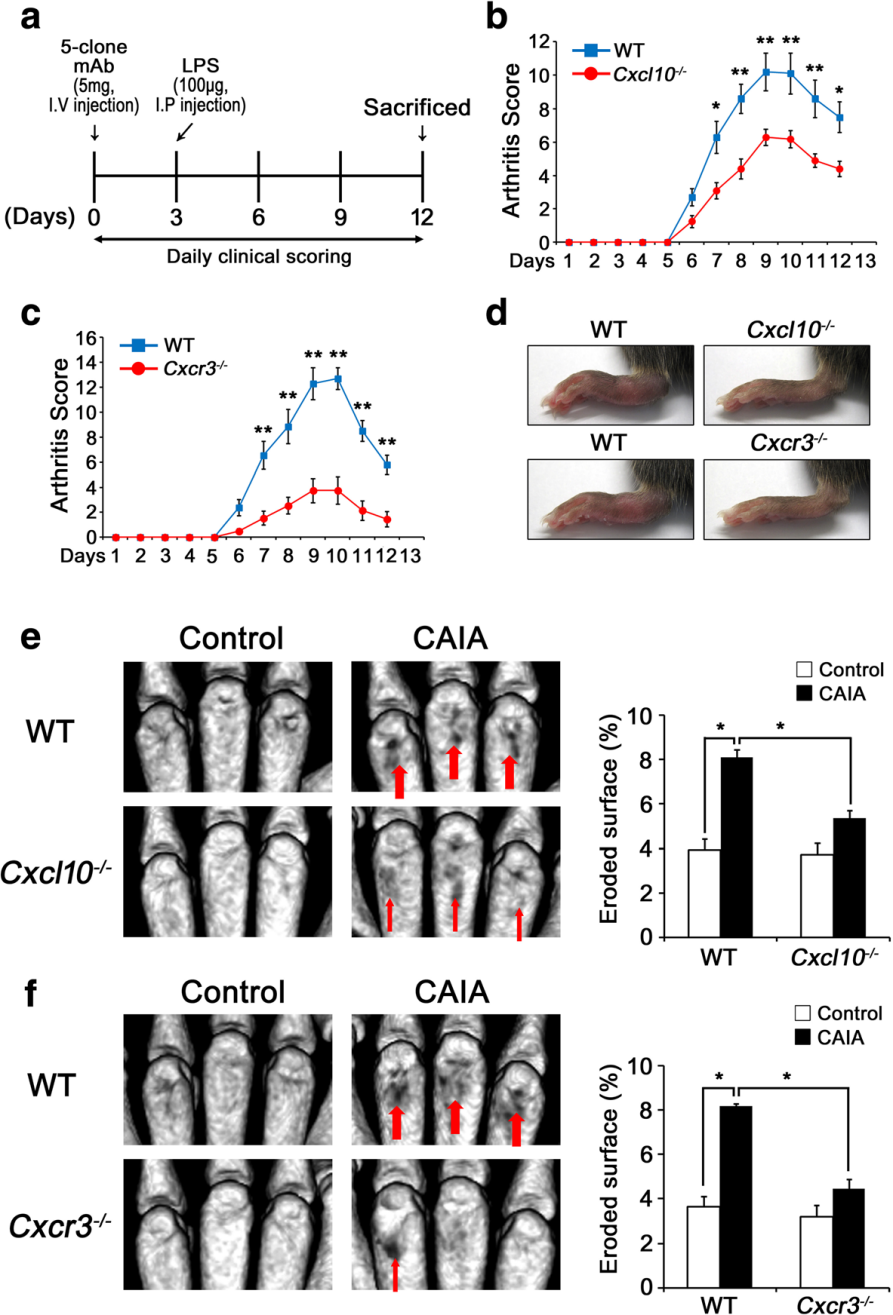
Amelioration of CAIA development in *Cxcl10* ^*–/–*^ and *Cxcr3* ^*–/–*^ mice. **a** Experimental design of CAIA. WT, *Cxcl10* ^*–/–*^, and *Cxcr3* ^*–/–*^ mice were subjected to collagen type II antibodies + LPS (CAIA) or LPS injection (control). **b**–**d** Development of CAIA was monitored for 12 days, and arthritic scores were assessed by the degree of swelling in each paw (scored 0–3). Arthritic scores in WT and *Cxcl10* ^***–/–***^ mice (**b**, *n* = 5 each) and WT and *Cxcr3* ^*–/–*^ mice (**c**, *n* = 5 each) subjected to CAIA. Data represent mean ± SEM. *P < 0.01; **P < 0.001 vs WT at each time point by two-way repeated-measures ANOVA followed by Bonferroni’s test. Representative photographs of CAIA-induced paw swelling on day 9 (**d**). **e**, **f** Representative micro-CT images and quantification of bone surface erosion in the hind paws from WT and *Cxcl10* ^*–/–*^ mice (**e**; *n* = 5 each group) and WT and *Cxcr3* ^*–/–*^ mice (**f**, *n* = 5 each group) on day 12. *Arrows* indicate bone erosions. Data represent mean ± SEM. *P < 0.001 by one-way ANOVA followed by Dunnett’s test. *LPS* lipopolysaccharide, *WT* wild-type, *CXCL10* C-X-C motif chemokine 10, *Cxcr3* CXC chemokine receptor 3, *CAIA* collagen antibody-induced arthritis

To determine the role of CXCL10 signaling in the development of arthritis, we used the CAIA model in mice lacking *Cxcl10* ^*–/–*^ and *Cxcr3* ^*–/–*^. As shown in Fig. 3a, LPS was used as a booster after injection of collagen type II antibodies to induce severe arthritis in the CAIA model. Because LPS is a specific ligand for TLR4, we excluded *Tlr4* ^–/–^ mice in this study. In C57BL/6 WT mice, clinical signs of arthritis were first observed 6 days after injection of collagen type II antibodies and reached a maximum on days 9–10. *Cxcl10* ^*–/–*^ and *Cxcr3* ^*–/–*^ mice developed less severe arthritis than WT mice (Fig. 3b, c). Consistent with the results of clinical scores, both *Cxcl10* ^*–/–*^ and *Cxcr3* ^*–/–*^ mice had attenuated paw swelling compared with WT mice (Fig. 3d). Next, we performed three-dimensional reconstruction and analysis of paws by micro-CT to check bone erosion status. Micro-CT scans revealed erosive bone lesions and an increase in eroded surface per total bone surface following CAIA, which were significantly decreased in *Cxcl10* ^*–/–*^ and *Cxcr3* ^*–/–*^ mice compared with WT mice (Fig. 3e, f).

**Fig. 4:**
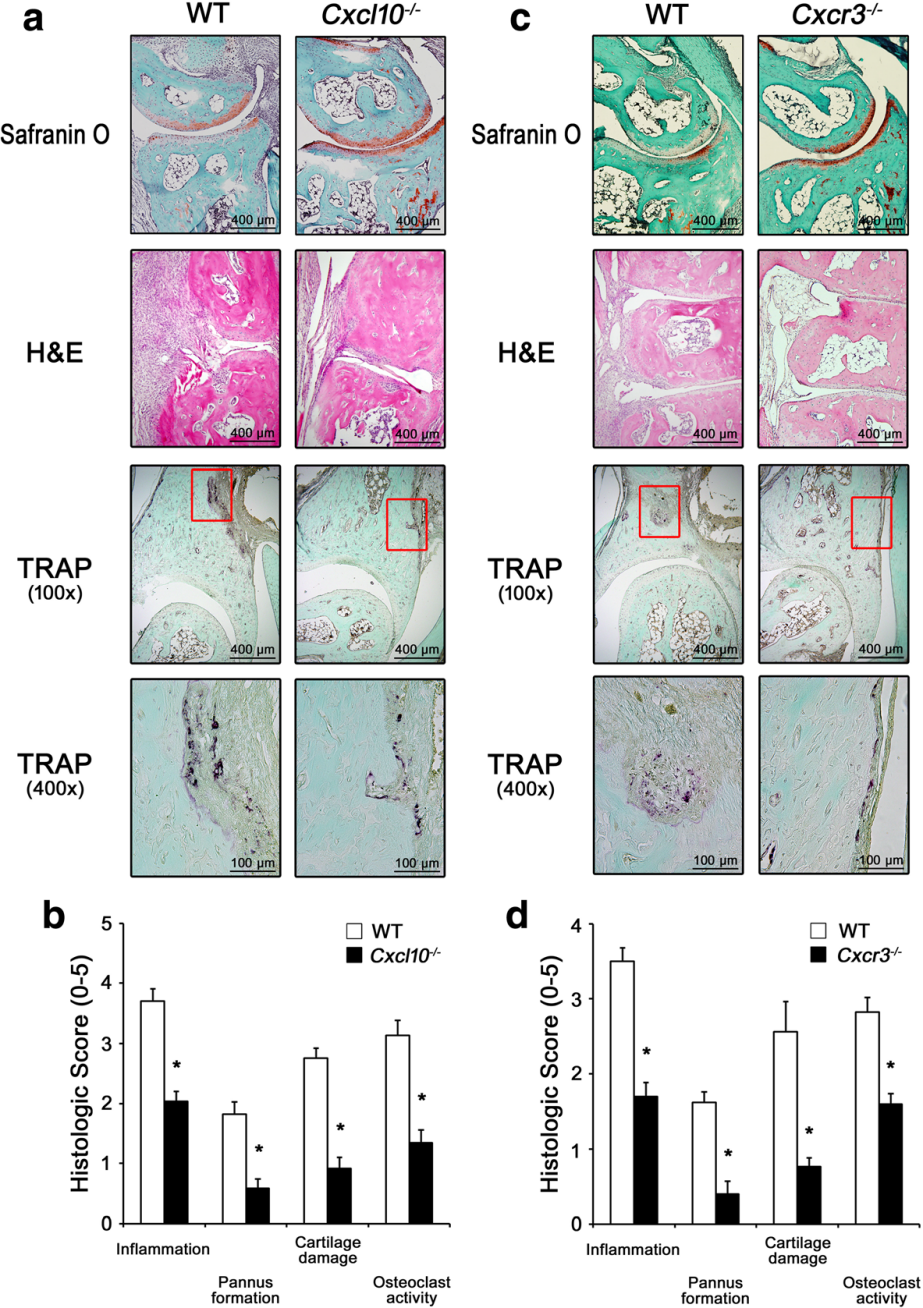
Histological analysis of arthritic lesions in *Cxcl10* ^*–/–*^ and *Cxcr3* ^*–/–*^ mice with CAIA. Tissue sections were obtained from paw joints of the same mice as in Fig. 3 on day 12 after arthritic induction. Representative images of Safranin O, H&E, and TRAP staining (**a**, **c**) and quantification of histological analysis (**b**, **d**) of joint tissue sections from WT and *Cxcl10* ^*–/–*^ mice (**a**, **b**) and WT and *Cxcr3* ^*–/–*^ mice (**c**, **d**) with CAIA. *Fourth panel* (**a**, **c**) is a higher magnification image of the boxed area in the *third panel. Scale bar*, 400 μm (*first–third panels*) and 100 μm (*fourth panel*). Data represent mean ± SEM of results in five samples of staining (*n* = 5). *P < 0.001 vs WT mice by unpaired Student’s *t* test. *WT* wild-type, *CXCL10* C-X-C motif chemokine 10, *Cxcr3* CXC chemokine receptor 3, *H&E* hematoxylin and eosin, *TRAP* tartrate-resistant acid phosphatase (Color figure online)

To accurately evaluate the degree of inflammation, pannus formation, and cartilage destruction, joint tissue sections were examined using a histopathologic scoring system [26, 27]. Histopathologic studies also revealed milder inflammation, pannus formation, and cartilage degeneration following CAIA in *Cxcl10* ^*–/–*^ and *Cxcr3* ^*–/–*^ mice than in WT mice (Fig. 4a–d). In order to detect osteoclasts, we performed TRAP staining. In line with the results of micro-CT analysis, lower osteoclast activity was observed in *Cxcl10* ^*–/–*^ and *Cxcr3* ^*–/–*^ mice than in WT mice (Fig. 4a–d).

Taken together, these results demonstrate that the clinically observed amelioration of arthritis in *Cxcl10* ^*–/–*^ and *Cxcr3* ^*–/–*^ mice was reflected by suppression of joint inflammation and destruction of bone and cartilage.

### *Cxcl10*^*–/–*^ and *Cxcr3*^*–/–*^ mice with CAIA have decreased F4/80^+^ macrophages and CD4^+^ T-cell accumulation in arthritic joints and serum concentrations of osteoclastogenic cytokines

**Fig. 5:**
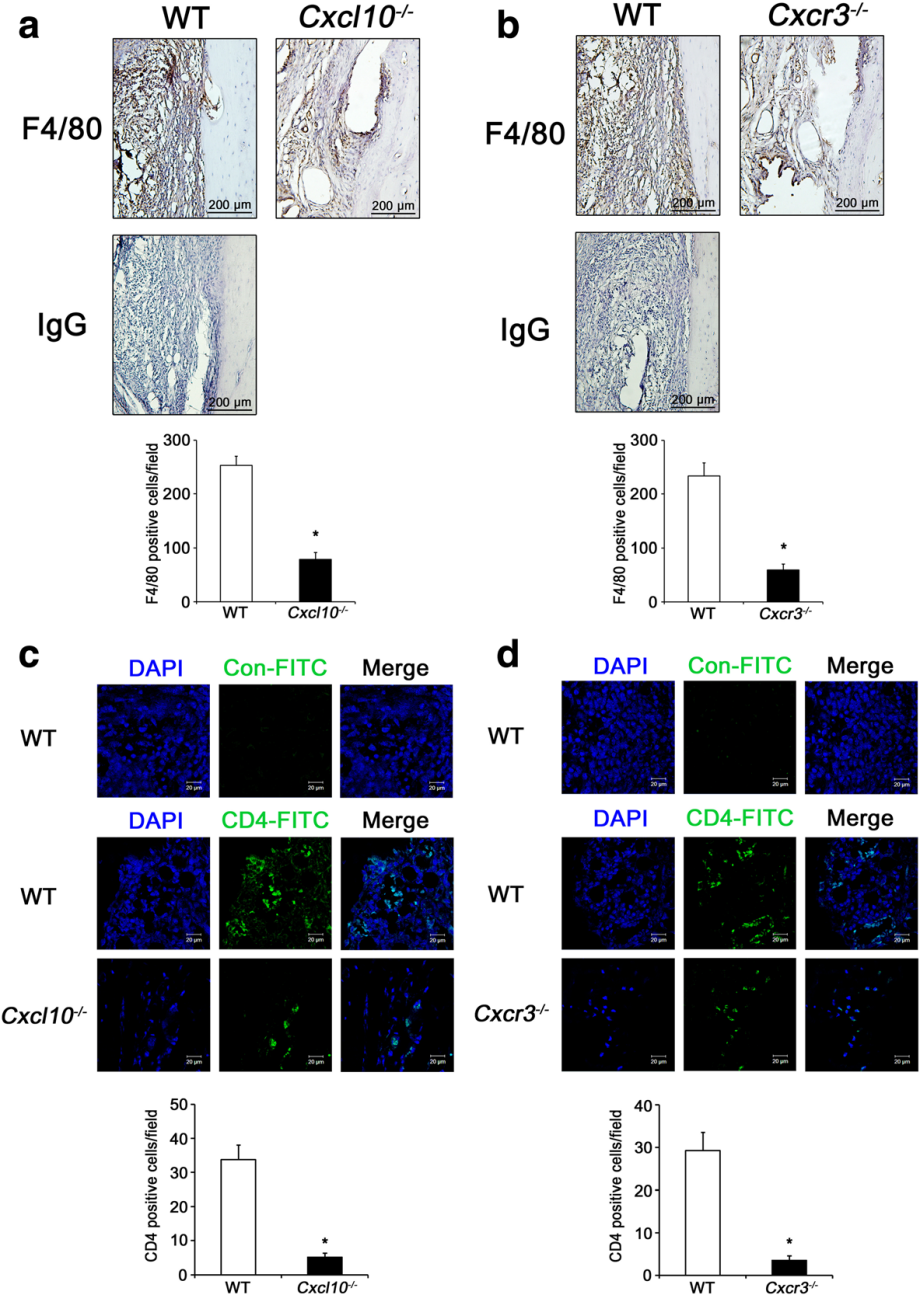
Reduction of macrophages and CD4^+^ T cells accumulation in arthritic joints of *Cxcl10* ^*–/–*^ and *Cxcr3* ^*–/–*^ mice with CAIA. **a**, **b** Joint tissue sections from WT, *Cxcl10* ^*–/–*^ (**a**), and *Cxcr3* ^*–/–*^ (**b**) mice with CAIA used in Fig. 4 were stained with an IgG or an anti-F4/80 antibody. Representative images of F4/80 staining of joint tissue sections from each group (*top panel*) and quantification of F4/80-positive cells (*bottom panel*). IgG, negative control. *Scale bar*, 200 μm. Data represent mean ± SEM of results in five samples (*n* = 5). *P < 0.001 vs WT mice by unpaired Student’s *t* test. **c**, **d** Joint tissue sections from WT, *Cxcl10* ^*–/–*^ (**c**), and *Cxcr3* ^*–/–*^ (**d**) mice with CAIA used in Fig. 4 were stained with a control-FITC or an anti-CD4-FITC antibody. Representative images of CD4-FITC staining of joint tissue sections from each group (*second and third topmost panels*) and quantification of CD4-positive cells (*bottom panel*). Con-FITC, negative control. *Scale bar*, 20 μm. Data represent mean ± SEM of results in five samples (*n* = 5). *P < 0.001 vs WT mice by unpaired Student’s *t* test. *WT* wild-type, *CXCL10* C-X-C motif chemokine 10, *Cxcr3* CXC chemokine receptor 3, *IgG* immunoglobulin G, *DAPI* 4′,6-diamidino-2-phenylindole, *FITC* fluorescein isothiocyanate

Next, we carried out immunohistochemistry with an anti-F4/80 antibody and immunohistofluorescence with an anti-CD4-FITC antibody to check the extent of F4/80^+^ macrophages and CD4^+^ T-cell infiltration into the synovium, respectively. A significant reduction in both F4/80^+^ macrophages and CD4^+^ T-cell infiltration was observed in *Cxcl10* ^*–/–*^ and *Cxcr3* ^*–/–*^ mice compared with WT mice under the CAIA condition (Fig. 5a–d).

**Fig. 6:**
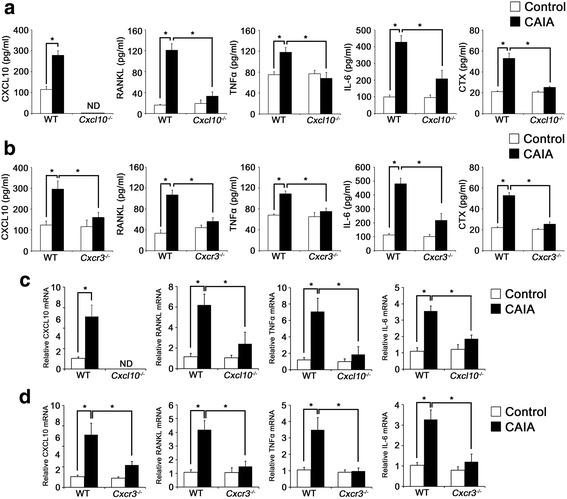
Reduction of osteoclastogenic cytokine levels in serum and spleen of *Cxcl10* ^*–/–*^ and *Cxcr3* ^*–/–*^ mice with CAIA. **a**, **b** Serum was collected from the same WT, *Cxcl10* ^*–/–*^ (**a**), and *Cxcr3* ^*–/–*^ (**b**) mice as in Fig. 3 on day 12. Serum levels of CXCL10, RANKL, TNFα, IL-6, and CTX were measured by ELISA. Data represent mean ± SEM. *P < 0.001 by one-way ANOVA followed by Dunnett’s test. **c**, **d** Spleens were collected from the same WT, *Cxcl10* ^*–/–*^ (**c**), and *Cxcr3* ^*–/–*^ (**d**) mice as in Fig. 3 on day 12. RANKL, TNFα, IL-6, and CXCL10 mRNA levels were analyzed by real-time PCR. Data represent mean ± SEM of results in five samples (*n* = 5). *P < 0.001 vs WT mice by unpaired Student’s *t* test. *WT* wild-type, *CXCL10* C-X-C motif chemokine 10, *Cxcr3* CXC chemokine receptor 3, *CAIA* collagen antibody-induced arthritis, *RANKL* receptor activator of nuclear factor kappa-B ligand, *TNFα* tumor necrosis factor α, *IL-6* interleukin 6, *CTX* C-terminal telopeptide, *ND* not detected

We have shown previously that CXCL10 levels are markedly increased in the inflamed joints and serum of CIA mice and that neutralization of CXCL10 by antibodies decreases CIA-induced elevation of RANKL and TNFα levels in serum [6]. Similar to the previous findings, C57BL/6 WT mice with CAIA exhibited a significant elevation of CXCL10, RANKL, TNFα, and IL-6 levels in serum compared with control mice. However, the CAIA-induced protein levels of RANKL, TNFα, and IL-6 in serum were markedly suppressed in *Cxcl10* ^*–/–*^ and *Cxcr3* ^*–/–*^ mice compared with their WT counterparts (Fig. 6a, b). In addition, CAIA greatly induced the mRNA expression of CXCL10, RANKL, TNFα, and IL-6 in spleens from WT mice, but not in spleens from *Cxcl10* ^*–/–*^ and *Cxcr3* ^*–/–*^ mice (Fig. 6c, d). Interestingly, we also found that the elevated protein or mRNA expression of CXCL10 in serum or spleen from WT mice with CAIA is abrogated in *Cxcr3* ^–/–^ mice (Fig. 6b, d), suggesting that CXCR3 signaling may involve an amplification of its ligand CXCL10 in the CAIA model.

Because CAIA-induced osteoclast activity was significantly decreased in *Cxcl10* ^*–/–*^ and *Cxcr3* ^*–/–*^ mice (Fig. 4), we measured serum CTX levels, a marker of bone resorption. As shown in Fig. 6a, b, serum CTX levels were increased by CAIA in WT mice, which was greatly inhibited in *Cxcl10* ^*–/–*^ and *Cxcr3* ^*–/–*^ mice.

## Discussion

CXCL10 has been found to be highly expressed in the serum and synovial fluid of RA patients [15, 16]. Furthermore, previous studies have shown that pharmacologic blockade of CXCL10 signaling inhibits arthritis progression in animal models, mainly via inhibition of T-cell migration into the joint [6, 17, 35]. In the present study with *Cxcl10* ^*–/–*^ and *Cxcr3* ^*–/–*^ mice, we showed that the CXCL10–CXCR3 axis plays important roles in local and systemic inflammation in a CAIA model.

Infiltration of inflammatory cells into synovium is a crucial step in the progression of RA. The accumulation of inflammatory cells in the synovium mainly reflects migration rather than local proliferation [3]. CAIA is reported to be induced by infiltration of neutrophils and macrophages, which are the main effector cells in CAIA, into the inflamed joints [20, 21, 36]. Al-Banna et al. [37] showed that the accumulation of T cells in inflamed lymph nodes is highly CXCR3 dependent and that CXCR3 deficiency reduces the development of arthritis in a CIA model. In addition, Mohan and Issekutz [17] showed that blockade of CXCR3 inhibits T-cell and neutrophil recruitment to inflamed joints and decreases the severity of adjuvant arthritis. In the present study, we found that CXCL10 stimulates migration of BMMs and CD4^+^ T cells through CXCR3-mediated ERK activation (Fig. 1). We also found that *Cxcl10* ^*–/–*^ and *Cxcr3* ^*–/–*^ mice showed less joint inflammation (Figs. 3 and 4) with reduced accumulation of F4/80^+^ macrophages and CD4^+^ T cells in the synovium (Fig. 5) compared with WT mice. Taken together, these results suggest that the CXCL10–CXCR3–ERK pathway may play a role in the accumulation of inflammatory cells during the development of arthritis.

It has been shown that inflammatory cytokine-expressing CD4^+^ T cells in the spleen and RANKL-expressing CD4^+^ T cells in blood are increased in the CAIA model [38]. In addition, inflammatory cytokine levels are systemically elevated in blood in CAIA mice [39]. Consistent with these reports, the protein and mRNA levels of RANKL, TNFα, IL-6, and CXCL10 in serum or spleens were elevated in WT mice with CAIA; however, elevation of these cytokines was abrogated in *Cxcl10* ^*–/–*^ and *Cxcr3* ^*–/–*^ mice (Fig. 6). We also found that CXCL10 stimulated the expression of RANKL, TNFα, and IL-6 in CD4^+^ T cells in a CXCR3-dependent and TLR4-dependent manner (Fig. 2a, b). Enhanced levels of RANKL and TNFα play an essential role in inflammation and mediate cartilage and bone destruction in RA [2, 40, 41]. CD4^+^ T cells are considered one of the major sources of inflammatory cytokines, especially of soluble RANKL, in arthritis. In fact, activated CD4^+^ T cells expressing soluble RANKL have been shown to cause severe joint inflammation and bone and cartilage destruction in adjuvant arthritis [42]. It is thus likely that CXCL10 signaling contributes to CAIA-induced RANKL production at least in part through CD4^+^ T cells in vivo. Recently, Grahnemo et al. [24] showed that CAIA is associated with an increased number of osteoclasts and trabecular bone loss. In the present study, CAIA-induced destruction of bone and cartilage in inflamed joints (Figs. 3 and 4) and elevation of CTX levels in serum (Fig. 6a, b) were significantly reduced in *Cxcl10* ^*–/–*^ and *Cxcr3* ^*–/–*^ mice. Collectively, these findings suggest that the elevation of CXCL10 signaling increases the production of cytokines including RANKL, TNFα, and IL-6, leading to destruction of bone and cartilage during the development of CAIA.

The RANKL gene promoter region in CD4^+^ T cells contains several recognition sequences for transcription factors, including NFAT, c-Fos, and NF-κB [31]. Wang et al. [32] have shown that intracellular calcineurin signaling downstream of T-cell receptor activation is critical for RANKL expression, which suggests that calcineurin-dependent NFAT activation may be involved in RANKL expression in CD4^+^ T cells. Activated calcineurin signaling can induce autoamplification of NFATc1 through NFATc1-dependent NFATc1 gene transcription [33]. In the present study, we found that CXCL10 increases NFATc1 and RANKL expression in CD4^+^ T cells in a CXCR3-dependent and TLR4-dependent manner (Fig. 2). Treatment of CD4^+^ T cells with CsA, an inhibitor of calcineurin activity, suppressed CXCL10-induced NFATc1 and RANKL expression. Moreover, the impairment of CXCL10-induced RANKL expression in *Cxcr3* ^*–/–*^ and *Tlr4* ^*–/–*^ CD4^+^ T cells was greatly rescued by overexpression of CA-NFATc1 (Fig. 2g, h). Therefore, our results indicate that CXCL10 induces RANKL expression in CD4^+^ T cells via the activation of NFATc1 in a CXCR3-dependent and TLR4-dependent manner. Although it has been shown that TLR4 is involved in the pathophysiology of RA in CIA and K**/**BxN serum transfer animal models [43, 44], the functional role of TLR4 in CXCL10-mediated production of cytokines contributing to destruction of bone and cartilage during the development of RA remains to be elucidated.

It is well known that CXCR3 is the main receptor for CXCL10. In line with this fact, CXCL10-induced cell migration and cytokine production were completely abolished in BMMs and CD4^+^ T cells from *Cxcr3* ^*–/–*^ cells (Figs. 1 and 2). Interestingly, however, we found that CXCL10-induced cytokine production was also abolished in *Tlr4* ^*–/–*^ CD4^+^ T cells even though the CXCR3 expression was intact in these cells (Fig. 2). These results suggest that TLR4 is specifically required for CXCR3 signaling in CXCL10-induced cytokine production, but not in CXCL10-induced cell migration. It has been reported that TLR4 activation induces an upregulation of chemokine expression including CXCL10 [45]. Recently, we have reported that CXCL10 induces CXCL10 expression through CXCR3 [46]. The TLR4 activation-induced or CXCR3-mediated CXCL10 upregulation can exert important roles in arthritis progression through TLR4 and CXCR3, suggesting that it could form a positive feedback loop between CXCL10 and its receptors TLR4 and/or CXCR3. In addition, several studies have shown crosstalk between TLRs and GPCR signaling. Eskan et al. [47] showed that TLR4 and S1P receptors coordinate to enhance inflammatory cytokine production in epithelial cells. GPCR agonist has been shown to transactivate TLRs via mammalian neuraminidase-1 sialidase and matrix metalloproteinase-9 crosstalk [48]. Moreover, TLR signaling has been reported to regulate GPCR signals by altering the expression of regulator of G protein signaling proteins in inflammatory cells [49, 50]. However, analysis of how TLR4 cooperates with CXCR3 in CXCL10-induced cytokine production remains to be elucidated.

As mentioned earlier, several cells including macrophages, T cells, neutrophils, and fibroblast-like synoviocytes have been considered relevant components of RA progression. These cells in the inflamed joint or other organs have roles in secretion of proinflammatory cytokines/chemokines, synovitis, and synovial pannus formation, leading to chronic inflammation and joint destruction [3]. In the present study, we used global *Cxcl10* ^*–/–*^ and *Cxcr3* ^*–/–*^ mice to investigate the pathogenic roles of CXCL10 signaling in a CAIA model and obtained less inflammation, accumulation of F4/80^+^ macrophages and CD4^+^ T cells, cytokine production, cartilage degeneration, and bone destruction in those mice. Accumulating evidence has shown that neutrophil recruitment in the joints is reduced by blockade of CXCR3 [17] and that CXCL10/CXCR3 signaling regulates invasion of fibroblast-like synoviocytes [51]. Given these reports, we cannot rule out that defective CXCL10 signaling in those cells including neutrophil and fibroblast-like synoviocytes might contribute to the inhibitory results of arthritis progression seen in *Cxcl10* ^*–/–*^ and *Cxcr3* ^*–/–*^ mice in our study. Furthermore, it has been reported that the accumulation of T cells in inflamed lymph nodes is highly CXCR3 dependent, but not CCR4 dependent [37]. Thus, we speculated that decreased recruitment of T cells in other organs including inflamed lymph nodes, not only in the synovium, would be observed in *Cxcl10* ^*–/–*^ and *Cxcr3* ^*–/–*^ mice; however, this hypothesis remains to be elucidated.

Collectively, our study provides data on the functional roles and molecular mechanisms of CXCL10 signaling in local and systemic inflammatory processes in RA. A study from human phase II clinical trials using anti-CXCL10 monoclonal antibody (MDX-1100) in RA patients has shown an improved response rate on day 58 [52]. In the present study, reducing the clinical severity of CAIA was more effective in *Cxcr3* ^*–/–*^ mice than in *Cxcl10* ^*–/–*^ mice (Fig. 3b, c). This might be due to the presence of other CXCR3 ligands including CXCL9 and CXCL11. Indeed, CXCL9 has been shown to be increased in the serum of RA patients [16]. In addition, CAIA-induced elevation of CXCL10 levels was abrogated in *Cxcr3* ^*–/–*^ mice (Fig. 6b, d). Thus, these findings suggest that CXCR3 would be a more attractive therapeutic target for RA than CXCL10.

## Conclusions

CXCL10 increased cell migration via CXCR3, not TLR4. However, both the CXCR3 and TLR4 receptors were required for CXCL10 to stimulate cytokine production in CD4^+^ T cells. The activated calcineurin-dependent NFATc1 pathway was essential for CXCL10-induced RANKL expression in CD4^+^ T cells. Arthritic *Cxcl10* ^*–/–*^ and *Cxcr3* ^*–/–*^ mice showed milder bone destruction, serum cytokines, and cell infiltration into synovium. These results indicate that CXCL10 may exert pathogenic roles through CXCR3 or TLR4 during arthritis progression.

## Additional file

Additional file 1: Pathogenic Roles of CXCL10 Signaling through CXCR3 and TLR4. **Fig. S1.** Purity and viability of CD4^+^ T cells from WT, *Tlr4* ^*-/-*^, and *Cxcr3* ^*-/-*^ mice. **Fig. S2**. The effect of si-CXCR3 on CXCL10-mediated BMM migration. **Fig. S3**. The effect of si-CXCR3 on CXCL10-mediated migration and cytokines expression of CD4^+^ T cells. **Fig. S4.** The effect of PD98059 on CXCL10-mediated ERK phosphorylation. (PDF 425 kb)

## Abbreviations

ACK: Ammonium–chloride–potassium
BMM: Bone marrow-derived macrophage
CAIA: Collagen antibody-induced arthritis
CIA: Collagen-induced arthritis
CsA: Cyclosporin A
CTX: C-terminal telopeptide
CXCL10: C-X-C motif chemokine 10
CXCR3: Chemokine receptor 3
DAPI: 4′,6-Diamidino-2-phenylindole
EDTA: Ethylenediaminetetraacetic acid
ELISA: Enzyme-linked immunosorbent assay
FITC: Fluorescein isothiocyanate
H&E: Hematoxylin and eosin
IFN-γ: Interferon gamma
IL: Interleukin
LPS: Lipopolysaccharide
NFATc1: Nuclear factor of activated T cells, cytoplasmic 1
PBS: Phosphate-buffered saline
RA: Rheumatoid arthritis
RANKL: Receptor activator of nuclear factor kappa-B
TLR4: Toll-like receptor 4
TNFα: Tumor necrosis factor alpha
TRAP: Tartrate-resistant acid phosphatase
WT: Wild-type
