# Supplementary material for: Pathogenic roles of CXCL10 signaling through CXCR3 and TLR4 in macrophages and T cells: relevance for arthritis
Source: Arthritis Res Ther. 2017 Jul 19;19:163. doi: 10.1186/s13075-017-1353-6 (PMC5518115; doi:10.1186/s13075-017-1353-6)
Supplement: Additional file 1: — Pathogenic Roles of CXCL10 Signaling through CXCR3 and TLR4. Fig. S1. Purity and viability of CD4+ T cells from WT, Tlr4 -/-, and Cxcr3 -/- mice. Fig. S2. The effect of si-CXCR3 on CXCL10-mediated BMM migration. Fig. S3. The effect of si-CXCR3 on CXCL10-mediated migration and cytokines expression of CD4+ T cells. Fig. S4. The effect of PD98059 on CXCL10-mediated ERK phosphorylation. (PDF 425 kb) [file 13075_2017_1353_MOESM1_ESM.pdf]

Pathogenic Roles of CXCL10 Signaling through CXCR3 and TLR4

Jong-Ho Lee,[2,#] Bongjun Kim,[1,#] Won Jong Jin,[1] Hong-Hee Kim,[1] Hyunil Ha,[3,*] and Zang Hee Lee[1,*]

[1]Department of Cell and Developmental Biology, Dental Research Institute, School of Dentistry, Seoul National University, Seoul 110-749, Republic of Korea

#These authors contributed equally to this work.

*Corresponding Authors:

Zang Hee Lee, DDS, PhD

Department of Cell and Developmental Biology, School of Dentistry, Seoul National University, 28 Yeongon-dong, Jongno-gu, Seoul 110-749, Republic of Korea.

Tel: +82-02-740-8672, Fax: +82-02-747-6589, e-mail: zang1959@snu.ac.kr

Hyunil Ha, PhD

Clinical Research Division, Korea Institute of Oriental Medicine, 483 Expo-Ro, Yuseong-Gu, Daejeon 305-811, Republic of Korea.

Tel: +82-42-868-9367; Fax: +82-42-868-9668; E-mail: hyunil74@kiom.re.kr

Supplemental methods

Flow cytometry

All staining and washes were carried out in ice-cold fluorescence-activated cell sorting (FACS) buffer (PBS + 0.5% BSA + 2 mM ethylenediaminetetraacetic acid (EDTA) + 0.05% w/v sodium azide) and tubes incubated on ice to minimize antibody internalization. Non-specific binding was limited by incubation with 0.25 μg of an anti-CD16/CD32 (Fc blocking antibody 2.4G2, eBioscience, San Diego, California, USA) per 1 million cells in 100 μL for 30 minutes. Surface labelling was carried out for 60 minutes with optimal concentrations of the relevant antibodies as decided by prior antibody titrations. The antibodies used in this study include rat anti-mouse; -CD4 FITC (clone GK1.5, eBioscience, San Diego, California, USA). The rat igG2b K isotype control FITC (clone eB149/10H5, eBioscience, San Diego, California) was used to control staining specificity.

Cell proliferation assay

Cell proliferation assay was performed by using a CCK-8 kit (Cell Counting Kit-8, Dojindo Molecular Technology, Japan), following the manufacturer's instruction. In brief, $CD4^+$ T cells were seeded at the density of $5x10^3$ in 96-well plates and cultured in the presence or absence of IL-2 (20ng/ml) for 0, 1, 2, or 3 days. Thereafter, CCK-8 kit solution was added to each well. After 2 hours of incubation at 37 °C in 5% CO_2 incubator, absorbance was measured at 455 nm by using IMARK micro-plate absorbance reader (Bio-Rad Laboratories, CA).

Reverse transcription and real-time PCR analysis

Total RNA and cDNA were prepared as described in Methods. The following primer sets

were used: mouse IL-2 forward, 5'- GCTGTTGATGGACCTACAGGA-3', and reverse, 5'-ATCCTGGGGAGTTTCAGGTT-3'.

Gene silencing

For gene silencing experiments, the following siRNA duplexes were used: a mix of 5′-ATGTTGAACAAGGCGCCTGAdTdT-3′ and 5′- TCTAGCACTTGACGTTCACAdTdT-3′ for mouse CXCR3 (Sigma-Aldrich, St Louis, MO) and siRNA Universal Negative Control (Sigma-Aldrich, St Louis, MO) as a negative control. Cells were transfected with 100 nM concentrations of the indicated siRNA duplexes using HiPerFect transfection reagent (Qiagen, Valencia, CA) for 12 hours according to the manufacturer's instructions. After 24 hours, the cells were used for analyzing cell migration or gene expressions.

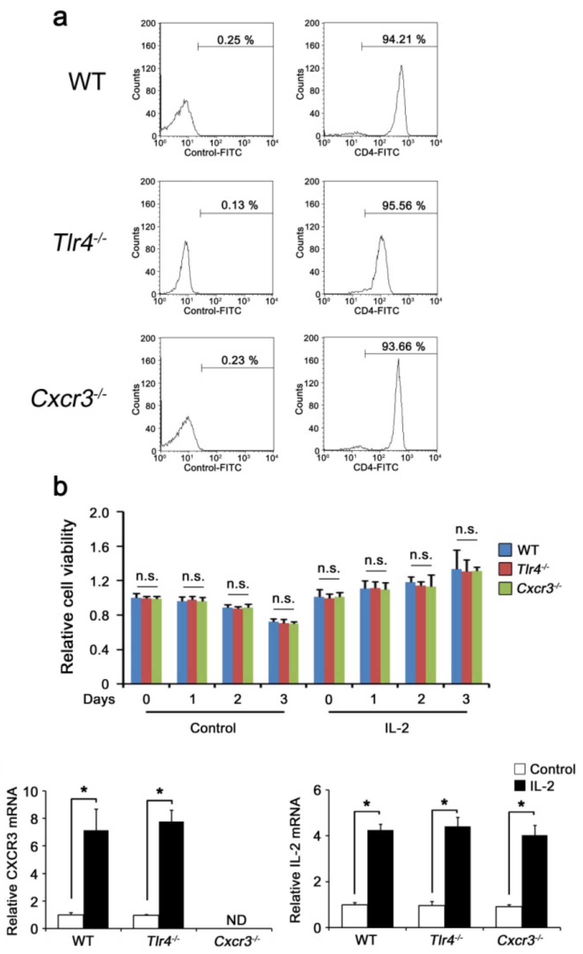

Fig. S1 Purity and viability of CD4$^+$ T cells from WT, *Tlr4$^{-/-}$*, and *Cxcr3$^{-/-}$* mice.

a CD4$^+$ T cells were isolated from wild-type (WT), *Tlr4$^{-/-}$*, and *Cxcr3$^{-/-}$* mice and then CD4$^+$ cells were analyzed by flow cytometry. The data are representative of three independent experiments.

b CD4$^+$ T cells from WT, *Tlr4$^{-/-}$*, and *Cxcr3$^{-/-}$* mice were cultured in the presence or absence of IL-2 (20ng/ml) for indicated times. Viability of the cells was measured using a CCK-8 kit.

c CD4$^+$ T cells from WT, *Tlr4$^{-/-}$*, and *Cxcr3$^{-/-}$* mice were isolated as described in Methods. The CD4$^+$ T cells were serum-starved and cultured in the presence or absence of IL-2 (20ng/ml) for 24 h. CXCR3 and IL-2 mRNA levels were analyzed by real-time PCR. The results shown are representative of three independent experiments (n=3), and the values are expressed as mean ± SD. *$P < 0.001$ by one-way ANOVA followed by Dunnett's test.

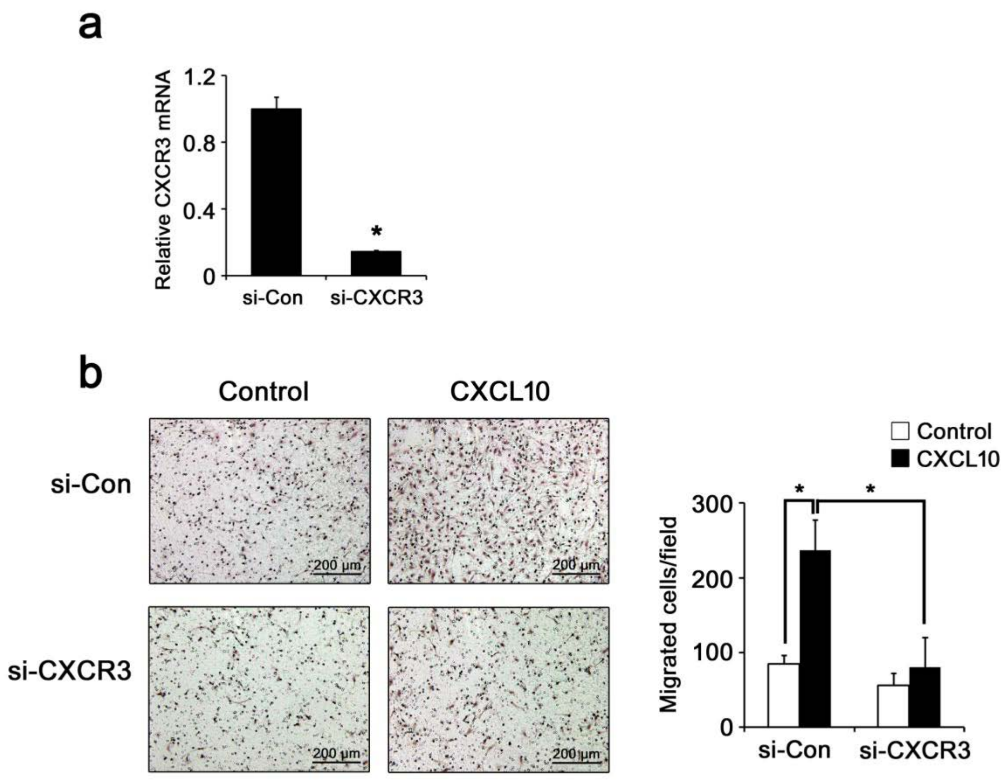

Fig. S2 The effect of si-CXCR3 on CXCL10-mediated BMM migration.

a BMMs were transfected with si-Control or si-CXCR3 for 12 h. After 24 h, CXCR3 mRNA levels were analyzed by real-time PCR.

b BMMs were transfected with si-Control or si-CXCR3 for 12 h. After 24 h, the BMMs were serum starved and cell migration in response to CXCL10 (100 ng/mL) was assessed in transwell chambers for 12 h. Representative images of migrated BMMs. (*left panel*; Scale bar, 200 μm) and number of migrated BMMs (*right panel*). The results shown are representative of three independent experiments (n=3), and the values are expressed as mean ± SD. *P < 0.001 by one-way ANOVA followed by Dunnett's test.

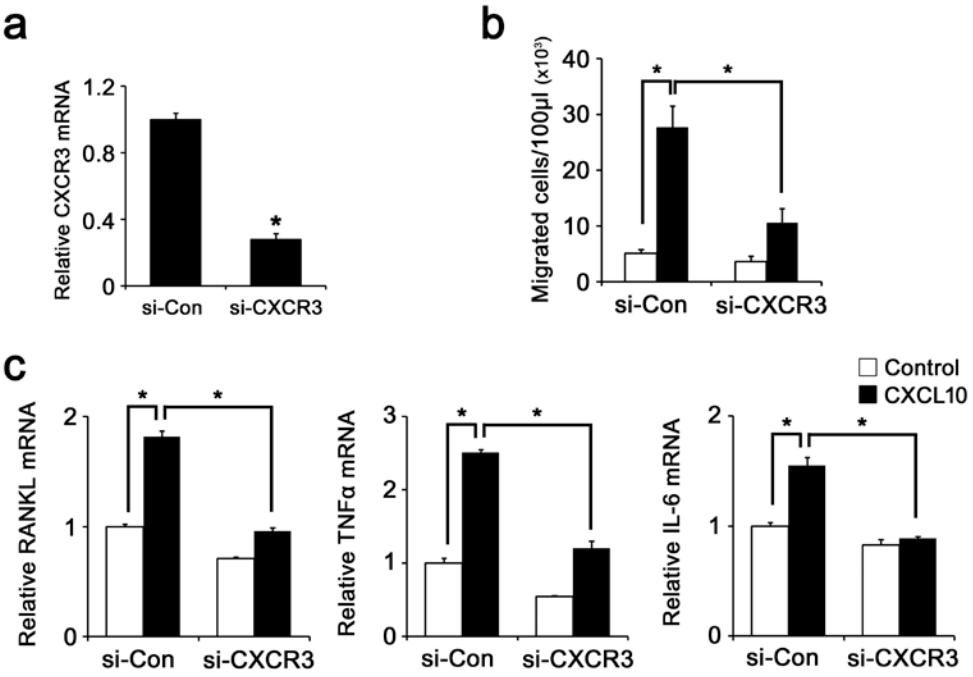

Fig. S3 The effect of si-CXCR3 on CXCL10-mediated migration and cytokines expression of $CD4^+$ T cells.

a $CD4^+$ T cells were transfected with si-Control or si-CXCR3 for 12 h. After 24 h, CXCR3 mRNA levels were analyzed by real-time PCR.

b $CD4^+$ T cells were transfected with si-Control or si-CXCR3 for 12 h. After 24 h of transfection, $CD4^+$ T cells were serum starved and cell migration in response to CXCL10 (100 ng/mL) was assessed in transwell chambers for 12 h.

c $CD4^+$ T cells were transfected with si-Control or si-CXCR3 for 12 h. After 24 h, $CD4^+$ T cells were serum starved and cultured in the presence or absence of CXCL10 (100 ng/mL) for 24 h. RANKL, TNFα, and IL-6 mRNA levels were analyzed by real-time PCR. The results shown are representative of three independent experiments (n=3), and the values are expressed as mean ± SD. *$P < 0.001$ by one-way ANOVA followed by Dunnett's test.

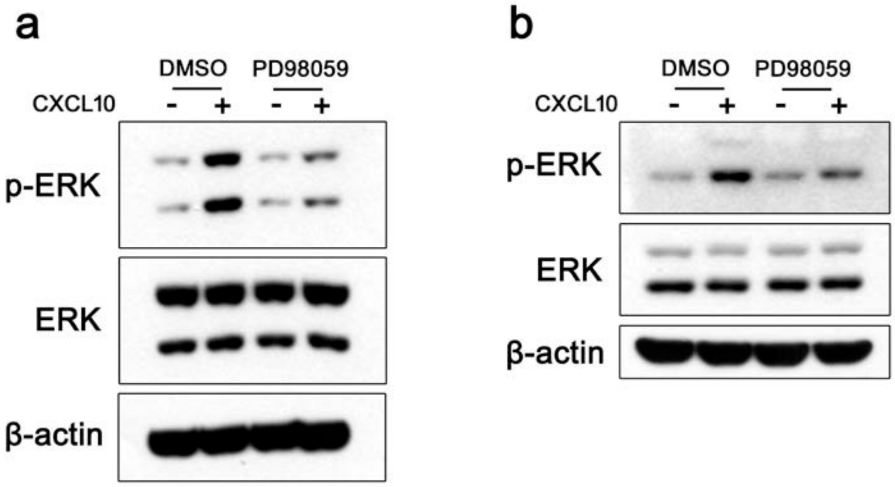

Fig. S4 The effect of PD98059 on CXCL10-mediated ERK phosphorylation.

a Serum-starved BMMs were preincubated with DMSO or 10 μM PD98059 for 1h, and then stimulated with or without CXCL10 (100 ng/mL) for 5 min. Total cell lysates were immunoblotted with the indicated antibodies.

b Serum-starved $CD4^+$ T cells were preincubated with DMSO or 10 μM PD98059 for 1h, and stimulated with or without CXCL10 (100 ng/mL) for 5 min. Total cell lysates were immunoblotted with the indicated antibodies. The data are representative of three independent experiments (n=3).
